# Molecular cloning and characterization of the allatotropin precursor and receptor in the desert locust, *Schistocerca gregaria*

**DOI:** 10.3389/fnins.2015.00084

**Published:** 2015-03-12

**Authors:** Els Lismont, Rut Vleugels, Elisabeth Marchal, Liesbeth Badisco, Pieter Van Wielendaele, Cynthia Lenaerts, Sven Zels, Stephen S. Tobe, Jozef Vanden Broeck, Heleen Verlinden

**Affiliations:** ^1^Molecular Developmental Physiology and Signal Transduction, KU LeuvenLeuven, Belgium; ^2^Department of Cell and Systems Biology, University of TorontoToronto, ON, Canada

**Keywords:** GPCR, insect, juvenile hormone, neuropeptide, orexin, peptide, motility

## Abstract

Allatotropins (ATs) are pleiotropic neuropeptides initially isolated from the tobacco hornworm, *Manduca sexta*. In 2008, the first receptor for AT-like peptides (ATR) was characterized in *Bombyx mori*. Since then, ATRs have also been characterized in *M. sexta*, *Tribolium castaneum*, *Aedes aegypti* and *Bombus terrestris*. These receptors show sequence similarity to vertebrate orexin (ORX) receptors. When generating an EST-database of the desert locust (*Schistocerca gregaria*) central nervous system, we found cDNA sequences encoding the *Schgr*-AT precursor and a fragment of its putative receptor. This receptor cDNA has now been completed and functionally expressed in mammalian cell lines. Activation of this receptor, designated as *Schgr*-ATR, by *Schgr*-AT caused an increase in intracellular calcium ions, as well as cyclic AMP (cAMP), with an EC_50_ value in the nanomolar range. In addition, the transcript distribution of both the *Schgr*-AT precursor and *Schgr*-ATR was investigated by means of quantitative real-time PCR. Moreover, we found more evidence for the myotropic and allatostimulatory actions of *Schgr*-AT in the desert locust. These data are discussed and situated in a broader context by comparison with literature data on AT and ATR in insects.

## Introduction

Allatotropin (AT) was originally identified as an amidated tridecapeptide isolated from the nervous system of the lepidopteran *Manduca sexta*. It was named after its first known biological function, namely the ability to stimulate juvenile hormone (JH) biosynthesis in the *corpora allata* (CA) *in vitro* (Kataoka et al., 1989). Most ATs have a conserved C-terminal pentapeptide that consists of a TARGFa motif although the hymenopteran AT has an exceptional TAYGFa C-terminal (Veenstra et al., 2012). There are also AT-like peptides (ATLs) that contain more variation in their C-terminal motif but they can elicit allatotropic activity as well (Lee et al., 2002). ATs have been isolated from numerous arthropod species, identified from the sequences of cloned genes, or deduced *in silico* from nucleotide sequence databases (Elekonich and Horodyski, 2003; Weaver and Audsley, 2009; Egekwu et al., 2014). Despite its widespread appearance in numerous insects, neither the AT precursor gene nor the AT receptor gene have been identified in *Drosophila melanogaster* or in any other members of this genus (Hewes and Taghert, 2001; Vanden Broeck, 2001). Related peptides have been reported in other phyla beyond Arthropoda. These were isolated in mollusks (Harada et al., 1993; Li et al., 1993; Veenstra, 2010), flatworms (Adami et al., 2011) and annelids (Ukena et al., 1995; Veenstra, 2011), and recent phylogenetic analysis also showed the presence of this peptidergic system in other protostomes, but not in nematodes, and in some deuterostomes (Mirabeau and Joly, 2013).

AT has pleiotropic functions in a variety of insect species. It stimulates visceral muscle activity (Paemen et al., 1991; Duve et al., 1999, 2000), heart activity (Veenstra et al., 1994), ventral diaphragm oscillation (Koladich et al., 2002), plays a role in the photic entrainment of the circadian clock (Petri et al., 2002), controls the release of digestive enzymes in the midgut (Lwalaba et al., 2010), inhibits active ion transport in the midgut (Lee et al., 1998) and stimulates the secretion of saliva and the contractions of the muscles surrounding the salivary glands (Masood and Orchard, 2014). In *Culex pipiens*, ovarian development was arrested when nondiapausing females were injected with AT dsRNA immediately after adult eclosion, mimicking the diapausing phenotype (Kang et al., 2014). Furthermore, in *T. castaneum*, disrupted adult development and fecundity was observed after injections of AT dsRNA in young pupae (Abdel-latief and Hoffmann, 2014).

It has been suggested that the ancestral role for this peptide family is related to its myotropic role, while the stimulation of JH biosynthesis evolved secondarily in some insect groups (Elekonich and Horodyski, 2003). The myotropic activity of AT on the gut was also suggested to be important for feeding, since gut contractions are necessary to allow food motility and the flow of digestive enzymes (Oeh et al., 2001; Audsley and Weaver, 2009; Nagata et al., 2012).

ATs exert effects on their cellular targets by binding to receptors with high affinity binding sites that are members of the family of rhodopsin-like G protein-coupled receptors (GPCRs). The AT receptors (ATRs) are orthologous to vertebrate orexin/hypocretin receptors. To date, five ATRs have been characterized; namely the neuropeptide 16 receptor in *Bombyx mori* (Yamanaka et al., 2008), the receptors of *M. sexta* (Horodyski et al., 2011), *Tribolium castaneum* (Vuerinckx et al., 2011), *Aedes aegypti* (Nouzova et al., 2012) and *Bombus terrestris* (Verlinden et al., 2013). The first three were dose-dependently activated by *Manse*-AT. The *T. castaneum* receptor was also activated by *Schgr*-AT (which is identical to *Lom*-AG-MT1, the AT from *Locusta migratoria*) and by an endogenous AT-like peptide (*Trica*-ATL) predicted from the *Tribolium* genome (Vuerinckx et al., 2011). The ATR receptor of *B. terrestris* also responded to *Manse-AT*, *Schgr*-AT, and *Trica*-ATL, but much higher concentrations were needed for generating these pharmacological effects (Verlinden et al., 2013). Activation of these AT(L) receptors resulted in an elevation of intracellular calcium and cAMP concentrations (Horodyski et al., 2011; Vuerinckx et al., 2011; Verlinden et al., 2013).

ATR-like receptor genes can also be found in the genomes of the mosquitoes *Anopheles gambiae* and *Culex quinquefasciatus*, the pea aphid *Acyrthosiphon pisum*, the kissing bug *Rhodnius prolixus*, the monarch butterfly *Danaus plexippus*, the jewel wasp *Nasonia vitripennis*, the honey bee *Apis mellifera*, the alfalfa leafcutter bee *Megachile rotundata*; the ant species *Harpegnathos saltator*, *Acromyrmex echinatior* and *Solenopsis invicta* and various other insect species (Caers et al., 2012 and unpublished BLAST analysis).

In *Locusta migratoria* a member of the AT family was first identified as the accessory gland myotropin 1, since it was isolated from the male accessory glands and shown to stimulate contractility of the locust oviduct (Paemen et al., 1991, 1992). In *S. gregaria*, AT was found in the brain (protocerebrum, antennal lobes, and tritocerebrum), the circumoesophageal connectives, the suboesophageal ganglion (SOG), the stomatogastric nervous system and all thoracic and abdominal ganglia. No mass peak corresponding to AT was found in the locust *corpora cardiaca* (CC) or retrocerebral complex (Homberg et al., 2004; Clynen and Schoofs, 2009).

We now complement the data obtained in the above mentioned lepidopteran, coleopteran, dipteran and hymenopteran species with a quantitative analysis of the AT precursor and receptor transcripts in different tissues of a representative of the hemimetabolous insects, *S. gregaria*. In addition, we show more evidence for the allatostimulatory and myoactive roles of *Schgr*-AT in the desert locust.

## Materials and methods

### Rearing of animals

Gregarious desert locusts were reared under crowded conditions with controlled temperature (30 ± 1°C), light (14 h photoperiod) and ambient relative humidity (40–60%). The locusts were kept at high density (>200 locusts/cage) in special wooden cages and fed daily with fresh cabbage leaves supplemented with dry oat flakes. Mature females deposited their eggs in pots filled with a slightly moistened sterile sand mixture (7 parts sand, 3 parts peat, and 1 part water). After oviposition, these pots were collected once a week and set apart in empty cages, resulting in pools of hatched first instar hoppers, which differed by no more than 7 days in age. Depending on the experimental conditions, the locusts were further synchronized at the time of ecdysis (Badisco et al., 2011a; Marchal et al., 2011).

The breeding of solitarious desert locusts was performed under isolated conditions according to the method described by Hoste et al. (2002). Newly hatched hoppers were separated at the day of eclosion and were placed in individual containers. Temperature, light-dark photoperiods and food supply were similar for isolated-reared and crowded-reared locusts. All solitarious animals came from stocks that were reared under isolated conditions for at least three generations. To characterize the phase status of crowded-reared and isolated-reared locusts, morphometric measurements of femur length (F), caput width (C), and elytra (E) were performed (Dirsch, 1953). The F/C ratio increased, whereas the E/F ratio decreased in successive isolated-reared generations, indicating that individuals shifted toward the solitarious phase. The color and behavioral characteristics of crowded- and isolated-reared locusts were very typical for the gregarious and solitarious phase, respectively.

### Tissue collection

The locust tissues were dissected under a binocular microscope and immediately snap frozen in liquid nitrogen. In a first experiment, we collected three pools of each tissue (brain, optic lobes, *corpora cardiaca*, *corpora allata*, prothoracic gland, SOG, salivary gland, prothoracic ganglion, mesothoracic ganglion, metathoracic ganglion, gonads, fatbody, flight muscle, foregut, midgut, hindgut, Malpighian tubules, and male accessory gland) of 10 day old gregarious and solitarious males and females. The three pools consist of respectively 40, 10, and 10 animals. In a second tissue collection the abdominal ganglia were dissected from 10 day old gregarious animals. The first three (1–3) abdominal ganglia are fused to the metathoracic ganglion and the last four (8–11) are fused to each other and form the terminal abdominal ganglion (Burrows, 1996). Abdominal ganglia 4-5 and 6-7 were dissected together. The three pools males and females each consist of 10 animals. Until further processing, we stored all the tissue samples at −80°C to prevent degradation.

### RNA extraction and cDNA synthesis

The dissected pooled tissues (<100 mg) were collected in “MagNa Lyser green beads” 2.0 ml tubes (Roche). Semi-automated homogenization of these samples was performed in a MagNA Lyser® Instrument (Roche, Mannheim, Germany) according to the manufacturer's instructions. Total RNA was extracted from the tissue homogenate utilizing an “RNeasy® Lipid Tissue Mini Kit” (Qiagen, Germantown, MD) in combination with a DNase treatment (RNase-free DNase set, Qiagen) to eliminate potential genomic DNA contamination.

After verification of the RNA quantity and quality with the Nanodrop (Thermo Fisher Scientific Inc.), we transcribed the resulting total RNA using the SuperScript® III Reverse Transcriptase (Invitrogen™ Life Technologies, Carlsbad, CA) utilizing random hexamers and oligodT's as described in the protocol. Afterwards, the resulting cDNA was diluted tenfold.

### Molecular cloning

The *Schgr*-AT precursor and a partial fragment of the putative *Schgr*-ATR were found by scanning the EST database of *S. gregaria* (Badisco et al., 2011b). The sequence of the *Schgr*-AT precursor was confirmed by sequencing the plasmid that was used to produce the cDNA library. Additional sequence information of *Schgr-ATR* was obtained by 3′ and/or 5′ rapid amplification of cDNA-ends (RACE) using the “5′/3′ RACE Kit, 2nd Generation” (Roche) in combination with *Schgr*-ATR gene specific primers (see Supplementary Table 1).

cDNA covering the entire *Schgr*-ATR was amplified using a three step procedure. In the first step gene specific cDNA was made using the “Transcriptor High Fidelity cDNA Synthesis Kit” (Roche) and the gene specific primer 5′-TGATAAACATCACTCTGTAT-3′. Next, two PCR rounds were performed using the “Pwo DNA Polymerase” (Roche). The following cycle program was used twice: 94°C for 180 s followed by 30 cycles of 94°C for 45 s, 61°C for 60 s, 72°C for 120 s. The program ended at 4°C after a final elongation at 72°C for 10 min. In the first PCR round, the forward primer: 5′-TCTGCCCACAGTACATCCAA-3′ and the reverse primer: 5′-CACTCCACTAGCGACCACAA-3′ were used and in the second PCR round the forward primer: 5′-CACCATGACAGAGAACGAAAC-3′ and the reverse primer: 5′-GTTGCGGGTAAGGAGGTGT-3′ were used. After the first PCR round a PCR clean-up was performed using the “GenElute™ PCR Clean-Up Kit” (Sigma-Aldrich®).

The resulting PCR products were purified from a 1% agarose gel with the “GenElute™ Gel extraction Kit” (Sigma-Aldrich®). The *Schgr*-AT precursor was cloned in a “pCR™4-TOPO” vector (Invitrogen™) and the *Schgr*-ATR was cloned into a “pcDNA™3.1/V5-His TOPO®” vector (Invitrogen™) following the manufacturer's instructions. The vector was transformed into One Shot® TOP10 chemical competent *E. coli* cells (Invitrogen) and grown on LB agar plates (35 g/l; Sigma-Aldrich®) with ampicillin (10 mg/ml; Invitrogen™). Colonies with an insert were collected and grown in LB medium (Sigma-Aldrich®) with ampicillin (10 mg/ml). The plasmid was purified using the “GenElute™ Plasmid Miniprep Kit” (Sigma-Aldrich®). DNA Sequences were determined using the ABI PRISM 3130 Genetic Analyzer (Applied Biosystems®) following the protocol outlined in the “BigDye® Terminator v1.1 Cycle Sequencing Kit” (Applied Biosystems®).

### Phylogenetic and structural analysis

We compared the *Schgr*-ATR sequence with other insect ATR(-like) sequences. We aligned the following sequences with MUltiple Sequence Comparison by Log- Expectation (MUSCLE; Edgar, 2004): *S. gregaria* ATR (GenBank acc. no. **JN543509**), *M. sexta* ATR (GenBank acc. no. **ADX66344**), and *T. castaneum* ATR (GenBank acc. no. **XP_973738**). In addition, a phylogenetic tree was constructed with the neighbor-joining method, using the amino acid sequences [starting from transmembranic region (TM) 1 and ending with the TM7] from the *Schgr*-ATR, ATR-like receptors of insect organisms [the ones mentioned above, *B. terrestris* ATR (GenBank acc. no. **XP_003402490**), the *A. mellifera* ATR (GenBank acc. no. **XP_001120335**), *M. rotundata* ATR (GenBank acc. no. **XP_003708421**), *N. vitripennis* ATR (GenBank acc. no. **XP_008217710**), *R. prolixus* ATR (GenBank acc. no. **AHE41431**), *A. aegypti* ATR (GenBank acc. no. **AEN03789**), *B. mori* neuropeptide A5 and A16 receptor (GenBank acc. no. **NP_001127740** and **NP_001127714**), and *D. plexippus* ATR (GenBank acc. no. **EHJ74388**)] and the FMRFamide receptor of *D. melanogaster* (GenBank acc. no. **AAF47700**), to root the tree (MEGA software vs. 6; Tamura et al., 2013; 1000-fold bootstrap resampling).

### Cell culture and transfections

Pharmacological analyses were performed in Chinese hamster ovary (CHO) WTA11 cells stably co-expressing the bioluminescent protein apoaequorin (Brough and Shah, 2009) and the promiscuous G_α16_ subunit, which couples most agonist-induced GPCRs to the phospholipase C and calcium pathway, irrespective of their natural signaling cascade (Offermans and Simon, 1995; Milligan et al., 1996 cell lines were obtained from the Free University of Brussels and Euroscreen, Belgium). In subsequent experiments, CHO-PAM28 cells stably expressing apoaequorin, but not the promiscuous G_α16_, and human embryonic kidney (HEK) 293 cells (Invitrogen™) were used to measure the *Schgr*-ATR downstream signaling properties via calcium and cAMP, respectively.

CHO-WTA11 cells, CHO-PAM28 cells and HEK293 cells were cultured in monolayers in Dulbecco's Modified Eagles Medium nutrient mixture F12-Ham (DMEM/F12) (Sigma-Aldrich®) supplemented with 100 U/ml penicillin and 100 μg/ml streptomycin (Sigma-Aldrich®) to prevent bacterial contamination of gram-positive and gram-negative bacteria, respectively. The medium was also supplemented with 10% fetal bovine serum (Sigma-Aldrich®). For CHO-WTA11 cells, 250 mg/ml zeocin (Invitrogen™) was added to the medium, whereas for CHO-PAM28 cells, 5 μg/ml puromycin (Sigma-Aldrich®) was added to the medium. Puromycin and zeocin were initially used to select for cells stably expressing apoaequorin (CHO-PAM28) (Torfs et al., 2002), or both apoaequorin and G_α16_ (CHO-WTA11) (Blanpain et al., 1999) and are thus still used as additional antibiotics in the appropriate screens. All cells were maintained in an incubator at 37°C with a constant supply of 5% CO_2_.

Transfections with pcDNA™3.1-*Schgr*ATR or empty pcDNA™3.1 vector were carried out in T75 flasks at 60–80% confluency. Transfection medium for CHO cells was prepared using the Lipofectamine LTX Kit (Invitrogen™) with 2.5 ml DMEM/F12, 12.5 μ l PlusTM Reagent and 5 μ g vector construct (pcDNA™3.1-*Schgr*ATR or empty pcDNA™3.1 vector) in 5 ml polystyrene round-bottom tubes. After a 5 min incubation period at room temperature, 30 μ l LTX was added to the medium. After a further incubation period of 30 min at room temperature, the medium was removed from the cells and the DNA/LTX mix was added dropwise to the cells followed by 3 ml of fresh complete medium. The transfection medium used for HEK293 cells was similar to that used for CHO cells except that in addition to 4 μ g of pcDNA™3.1-*Schgr*ATR expression construct (or empty pcDNA3.1 vector plasmid), cells were co-transfected with 2 μ g of reporter CRE(6×)-Luc plasmid. This reporter plasmid contains six tandem repeats of a cAMP Responsive Element (CRE) in front of a minimum collate promoter and the ORF of luciferase (this reporter plasmid was also used in various other studies: for example Hearn et al., 2002; Johnson et al., 2004). Following transfection, cells were incubated overnight (37°C, 5% CO_2_), then 10 ml of cell medium was added followed by a second overnight incubation (37°C, 5% CO_2_). Ligand-induced changes in either, intracellular calcium or cAMP were then monitored in the cells as described below.

### Calcium reporter assay in CHO cells

CHO cells (CHO-WTA11 or CHO-PAM28) transfected with receptor expression construct (or empty vector) were detached with PBS, complemented with 0.2% EDTA (pH 8.0), and rinsed off the flask with DMEM/F12 without phenolred (Gibco®). The number of viable and nonviable cells was determined using a NucleoCounter® NC-100™ (Chemometic). The cells were pelleted for 4 min at 800 rpm and resuspended to a density of 5 × 10^6^ cells/ ml in sterile filtered bovine serum albumin (BSA) medium (DMEM/F12 with L-glutamine and 15 mM Hepes, without phenolred, supplemented with 0.1% BSA) and loaded with 5 μM Coelenterazine_h (Invitrogen™). Next, the cells were incubated for 4 h in the dark, at room temperature, while gently shaken to reconstitute the holo-enzyme aequorin. After a tenfold dilution 30 min prior to the screening, 50 μl cell solution was injected in the wells (~25000 cells/well) and exposed to *Schgr*-AT (GL Biochem, Shanghai, China) reconstituted in several dilutions in BSA medium distributed in the 96-well plate. In every row, BSA medium without potential ligands was placed in one well to serve as the blank for that row. The calcium response was recorded for 30 s on a multimode microplate reader Mithras LB 940 at a wavelength of 469 nm (Berthold Technologies). After 30 s, 0.1% Triton X-100 was added and the signal was measured for another 10 s. Triton X-100 is a non-ionic surfactant that will break the cell membranes so the total cellular calcium content can be measured. The total calcium signal (ligand + Triton X-100) is representative for the amount of cells present in the well. The response of each blank (small signal caused by cells ruptured by the injection in the wells) was subtracted from the luminescence obtained for wells within the same row. Calculations were made using the output file from the Microwin software (Berthold Technologies) in Excel (Microsoft). Further analysis was done in Excel and GraphPad Prism 5. An illustration of this assay is shown in Supplementary Figure S1.

### Cyclic AMP (cAMP) reporter assay in HEK293 cells

To monitor changes in intracellular cAMP levels, HEK293 cells co-transfected with receptor construct (or empty vector) and reporter gene plasmid (CRE_6×_-luciferase) were detached with PBS, complemented with 0.2% EDTA (pH 8.0), and rinsed off the flask with DMEM/F12 without phenolred (Gibco®). The number of viable and nonviable cells was determined using the NucleoCounter® NC-100™. The cells were pelleted for 4 min at 800 rpm and finally resuspended to a density of 1 × 10^6^ cells/ml in DMEM/F12 without phenolred, but containing 200 μM 3-isobutyl-1-methylxanthine (IBMX, Sigma–Aldrich®; 20 μl of 0.1 M IBMX in DMSO in 10 ml DMEM/F12) to prevent cAMP breakdown. Into each well of a white 96-well plate 50 μl of cell suspension (~50000 cells/well) was added to either 50 μl of DMEM/F-12 (with IBMX, but without phenolred) or DMEM/F-12 [with IBMX, without phenolred and containing 10 μM NKH-477 (a forskolin analog; used to enhance cAMP levels in the cells)] containing various concentrations of the allatotropin peptide (GL Biochem, Shanghai, China). In each row of the plate at least one well with only phenol red-free DMEM/F-12 with IBMX was measured. This well is used to calculate the blank level. The cells were then incubated (37°C, 5% CO_2_) for 3–4 h. Hereafter 100 μl of steadylite plus™ substrate (PerkinElmer) was added to each well using a multichannel pipette and the plate was incubated for 15 min in the dark. Finally, light emission resulting from the luciferase enzymatic activity was recorded at 0 s and 5 s on a multimode microplate reader Mithras LB 940 at a wavelength of 469 nm. The signal of the two measurements (0 and 5 s) was almost identical; an average of these two was used for further analysis. Results were analyzed by using Excel and Graphpad Prism 5 Software. An illustration of this assay is shown in Supplementary Figure S2.

### qRT-PCR study of transcript levels

Accurate normalization of the raw data was obtained by using the optimal combination of endogenous control genes. The best combination of reference genes was determined using GeNorm (Vandesompele et al., 2002) as described by Van Hiel et al. (2009) and Verlinden et al. (2010). The PCR reactions were performed in a 20 μl reaction volume following the manufacturer's instructions for the Fast SYBR® Green Master Mix (2×) (Applied Biosystems®). The final concentration of the primers was 500 nM. Primers for the endogenous controls, as well as for the *Schgr*-ATR and *Schgr*-AT target genes, were designed by means of the Primer Express® Software v2.0 (Applied Biosystems®). For primer sequences, see Table 1.

**Table 1:** **Oligonucleotide primers for qRT-PCR used in this study**.

	**Forward primer**	**Reverse primer**
Actin	5′-AATTACCATTGGTAACGAGCGATT-3′	5′-TGCTTCCATACCCAGGAATGA-3′
GAPDH	5′-GTCTGATGACAACAGTGCAT-3′	5′-GTCCATCACGCCACAACTTTC-3′
*Schgr*-AT	5′-ATGCAGAACAACCCGGAACT-3′	5′-CTGGTTAGCGTCCACGAACTT-3′
*Schgr*-ATR	5′-CGTCAACCCAGTCATCTACAACTT-3′	5′-TAGGCGCACGTCCAGAACA-3′

Abbreviations used: GAPDH, Glyceraldehyde-3-phosphate dehydrogenase; Schgr, Schistocerca gregaria.

To identify efficient primersets for the qRT-PCR, relative standard curves for the endogenous controls and the *Schgr*-ATR and *Schgr*-AT transcripts were generated with serial (10×) dilutions of a brain cDNA sample. Reactions were run in duplicate on a StepOne™ Plus System (ABI Prism, Applied Biosystems®) using the following thermal cycling profile: 95°C for 10 min, followed by 40 steps of 95°C for 3 s and 60°C for 30 s. After 40 cycles, samples were run for the dissociation protocol (i.e., melting curve analysis). Analysis of the dissociation curves of the different amplification products revealed a single melting peak. In addition, we analyzed the PCR products via agarose gel electrophoresis, showing the presence of a single band of the expected size for each transcript. Furthermore, sequencing of the PCR products ultimately confirmed the identity of the amplified DNA with their respective target sequences.

To study the transcript levels, we normalized, for each sample, the relative amount of transcript to the endogenous controls (Actin and GADPH) and calculated transcript levels relative to a calibrator sample (in this case, a mix of all measured tissues of males and females, gregarious and solitarious). The tissue, phase and sex distribution experiments were repeated three times with independent biological pools of adult *S. gregaria* tissues (40, 10, and 10 animals per pool). We detected no amplification of the fluorescent signal in any negative control sample, proving that the extraction procedure, including the DNase treatment, effectively removed genomic DNA from all the RNA samples and that there was no contamination. Statistical analysis was performed by means of SPSS (v17.0, SPSS Inc., Chicago, Illinois), using the Mann-Whitney U test for comparing two independent groups. A level of *P* < 0.05 was considered significant.

In a second qRT-PCR we studied the transcript levels in the abdominal ganglia in males and females of gregarious animals. The first three abdominal ganglia are fused to the metathoracic ganglion hence the same samples as in the first transcript study were used. For each sample the relative transcript amounts were normalized to the housekeeping genes coding for Actin and GADPH. The brain sample of the females (a sample from the first tissue distribution) was used as the calibrator sample.

### *Schgr*-AT bioassay

*Schgr*-AT was tested on an isolated gut preparation, as described by Schoofs et al. (1990). The midgut from a sexually mature male was ligated at both ends with strings by which the gut was suspended between the arm of a transducer and the bottom of a plastic chamber containing 2.5 ml *S. gregaria* saline (1L: 8.766 g NaCl; 0.188 g CaCl_2_; 0.746 g KCl; 0.407 g MgCl_2_; 0.336 g NaHCO_3_; 30.807 g sucrose; 1.892 g trehalose; pH 7.2) at room temperature (Supplementary Figure S3). The transducer monitored the contractions of the gut, which were visualized on a connected recorder (LKB 2210 recorder). When a constant rhythm of contractions was reached, 25 μl of 10 mM M *Schgr*-AT (GL Biochem, Shanghai, China) dissolved in saline (to reach a final concentration of one micromolar) or the same volume of saline without peptide was added to the chamber. In between two measurements the chamber was rinsed three times with saline; after this the constant contraction rhythm was restored.

### In vitro measurement of JH biosynthesis—radiochemical assay (RCA)

Rates of JH release and the JH content were measured by the *in vitro* radiochemical assay (RCA) originally described by Tobe and Pratt (Pratt and Tobe, 1974; Tobe and Pratt, 1974) and further discussed by Feyereisen and Tobe (1981) and Yagi and Tobe (2001). The RCA measures the rate of incorporation of the methyl group from [Methyl-14C] methionine (50 μM, 2.11 GBq/mmol, New England Nuclear Co.) into JH in isolated CA. CA were dissected out of the head of vitellogenic adult females, since it is known that their CA produce a high amount of JH (Tobe and Pratt, 1975). The dissected CA were directly transferred to conical glass vials holding 50 μl of radioactive TC199 medium [3 μCi/ml medium; lacking L-methionine, glucose, acetate and calcium (Gibco®, with Hank's sals, HEPES 25 mM)]. The individual CA were shaken at 30°C during the first incubated period of 3 h. Next, CA were transferred to fresh radioactive TC199 medium supplemented with 30 μM farnesoic acid (FA) to stimulate JH synthesis. 1 μM *Schgr*-AT was added to the experimental CA. 8 control CA and 9 *Schgr*-AT treated CA were tested. Incubation medium was extracted using 300 μl of iso-octane. The samples were vortexed and centrifuged for 10 min at 2000 rpm. The top 200 μl of the iso-octane layer was removed and put into scintillation vials containing 3 ml of scintillant (ICN) and measured in a liquid scintillation counter (Beckman, LS-6500).

The effect of *Schgr*-AT on the JH production of the CA was calculated by dividing the difference of the JH production during the second incubation and the JH production during the first incubation by the JH production during the second incubation. Significance was determined with a student's *t*-test in GraphPad Prism 5.

## Results

### Cloning and sequence analysis

**Figure 1:**
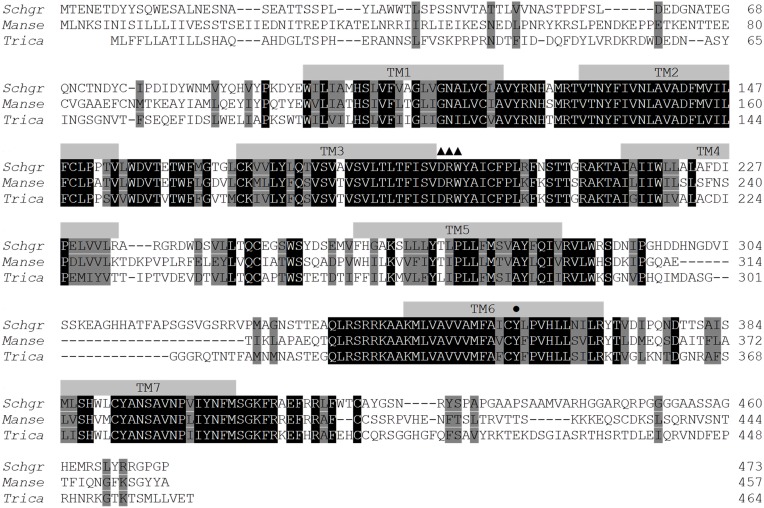
**Amino acid sequence of the *Schgr*-ATR (GenBank acc. no. JN543509) and homologous receptors from *Manduca sexta* (GenBank acc. no. ADX66344) and *Tribolium castaneum* (GenBank acc. no. XP_973738)**. The amino acid position is indicated at the right. Identical residues between the aligned sequences are highlighted in black, and conservatively substituted residues in gray. Dashes indicate gaps that were introduced to maximize homologies. Putative transmembrane regions (TM1-TM7) are indicated by gray bars. The position of the W (here changed to Y) (•) that is usually conserved in many rhodopsin-like GPCRs and the DRW motif (▲▲▲) are labeled.

**Figure 2:**
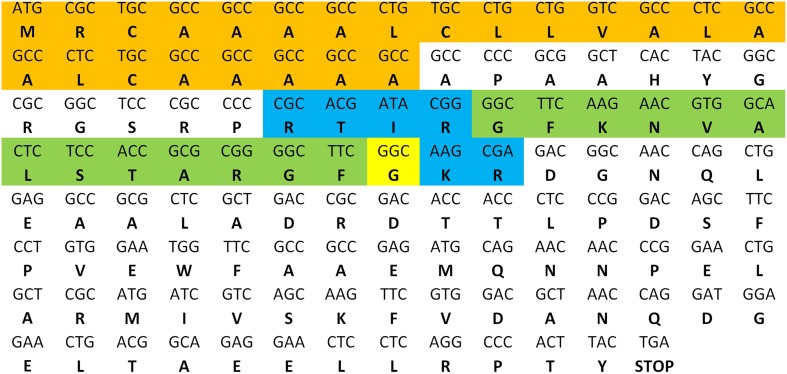
**Precursor sequence of *Schgr*-AT**. The sequence of AT is highlighted in green. The predicted signal peptide sequence is shown in orange and the recognition sites for proteolytic processing of the proneuropeptide are shown in blue. The G-residue predicted to be transformed into the C-terminal amide is shown in yellow.

As described in the *S. gregaria* EST paper (Badisco et al., 2011b), a partial fragment of an orexin 2 receptor-like/ ATR-like receptor and the *Schgr*-AT precursor are represented in the EST database. These sequences were confirmed by PCR, cloning, and sequencing. We further completed the sequence of the *Schgr*-ATR by rapid amplification of cDNA ends (RACE). The *Schgr*-ATR amino acid sequence is shown in Figure 1. The receptor belongs to the rhodopsin-like GPCRs and contains seven transmembrane domains [analyzed with a hidden Markov model for predicting transmembrane regions (Sonnhammer et al., 1998; Krogh et al., 2001)]. The sequence of the precursor *Schgr*-AT is displayed in Figure 2. The sequence contains a signal peptide predicted by SignalP 4.1 (Petersen et al., 2011) and two recognition motifs for proteolytic processing of the preproallatotropin. The G-residue at the C-terminal may be a substrate for peptidyl amidating monooxygenase (PAM) resulting in an amidated neuropeptide (Rouillé et al., 1995; Veenstra, 2000; Veenstra et al., 2012). The obtained nucleotide sequence of the *Schgr*-ATR fragment and the sequence of *Schgr*-AT have been submitted to the European Bioinformatics Institute (EBI) database (*Schgr-ATR*: GenBank accession no. **JN543509**; *Schgr-AT*: GenBank accession no. **KP233881**).

### Analysis of phylogenetic relationships

**Figure 3:**
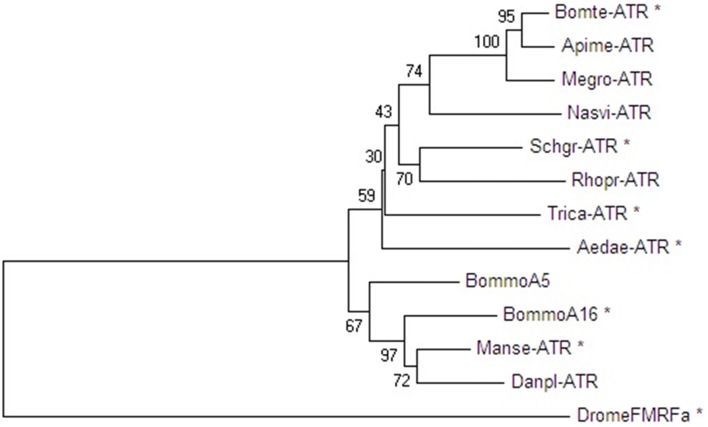
**Neighbor-joining tree of insect ATR-like receptors in dendrogram display with representative branch length**. Phylogenetic and molecular evolutionary analyses were conducted by using MEGA version 6. The FMRFamide-receptor of *D. melanogaster* (GenBank acc. no. **AAF47700**) was used as an outgroup to root the tree. Proteins marked with an asterisk were functionally characterized. Bootstrap-support values are based on 1000 replicates and are indicated on the nodes. The other GenBank accession numbers are: *S. gregaria* ATR (**JN543509**), *M. sexta* ATR (**ADX66344**), *T. castaneum* ATR (**XP_973738**), *B. terrestris* ATR (**XP_003402490**), *A. mellifera* ATR (**XP_001120335**), *M. rotundata* ATR (**XP_003708421**), *N. vitripennis* ATR (**XP_008217710**), *R. prolixus* ATR (**AHE41431**), *A. aegypti* ATR (**AEN03789**), *B. mori* neuropeptide A5 and A16 receptor (**NP_001127740** and **NP_001127714**), and *D. plexippus* ATR (**EHJ74388**).

Amino acid sequence comparisons between the *Schgr*-ATR and other insect ATR-like receptors show high overall amino acid similarity (identical and conservatively substituted residues; Figure 1). The amino acid sequences (TM1-7) of the ATR-like receptors and the FMRFamide receptor from *D. melanogaster*, to root the tree, were aligned with MUSCLE. A neighbor-joining tree was constructed using MEGA software with 1000-fold bootstrap resampling (Figure 3). The ATRs cluster together as compared to the root of the tree and the lepidopteran and hymenopteran ATRs cluster within their insect class. The overall insect phylogeny is however not respected in the tree. Bootstrap values already indicate that the power of some nodes is less as compared to the lepidopteran and hymenopteran cluster. Future characterization projects will hopefully result in more ATR sequences from diverse phylogenetic classes and will hopefully increase the overall power of phylogenetic studies.

### Functional activation of *Schgr*-ATR with AT

**Figure 4:**
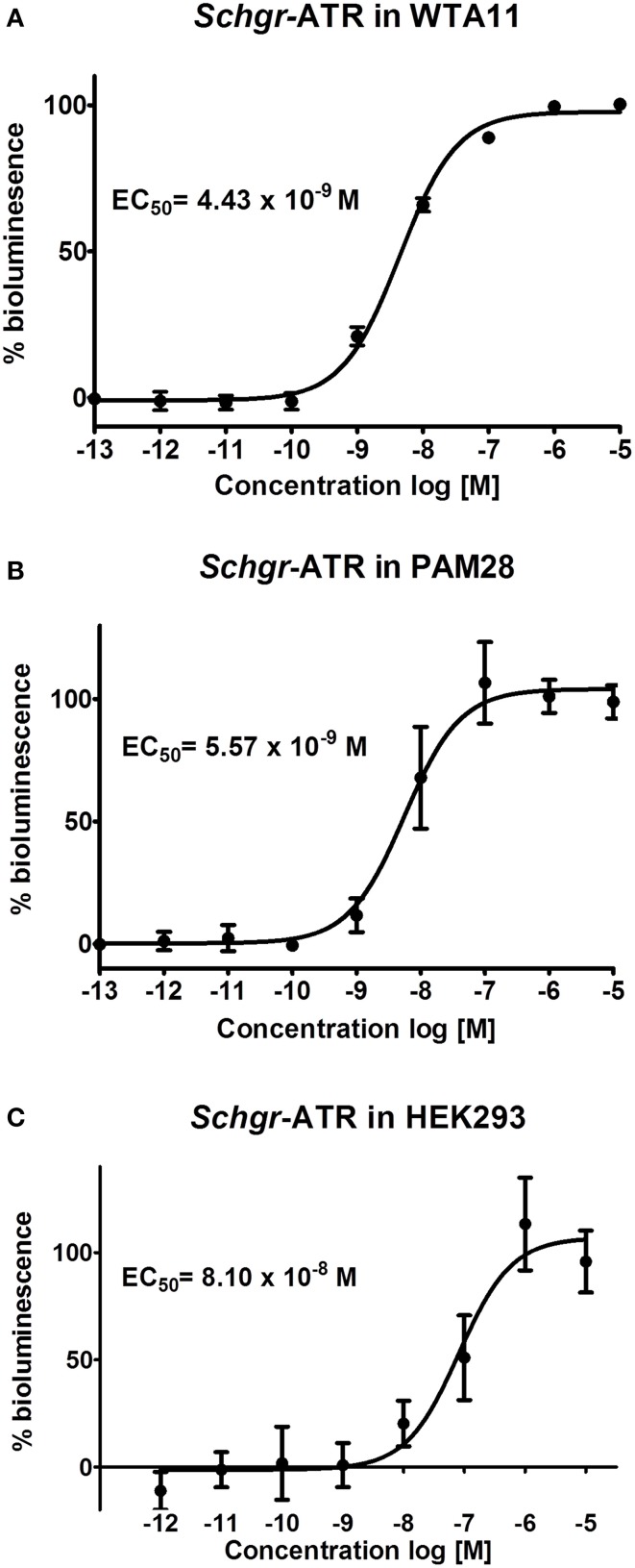
**Dose-response curve for bioluminescence responses induced in (A) CHO-WTA11-*Schgr*-ATR cells, (B) CHO-PAM28-*Schgr*-ATR cells, (C) HEK293- *Schgr* -ATR cells**. In all cell lines, the bioluminescence was measured in two independent transfections in triplicate and data are given in percentage (±S.D.) of the maximal response. The zero response level corresponds to treatment with BSA buffer only.

The *Schgr*-ATR was expressed in CHO-WTA11 cells, which express the promiscuous G_α16_ protein that can induce a calcium rise if an agonist (in this case *Schgr*-AT) binds to the receptor. *Schgr*-AT elicits a sigmoidal dose-dependent response with an EC_50_ value of 4.43 × 10^−9^ M (Figure 4A; logEC_50_ = −8.354 ± 0.025, mean ± SEM). To test if the receptor can induce a calcium rise, the receptor is expressed in CHO-PAM28 cells, which do not express the promiscuous G_α16_ protein. *Schgr*-AT clearly induced an intracellular calcium response with an EC_50_ value of 5.57 × 10^−9^ M (Figure 4B; logEC_50_ = −8.254 ± 0.067, mean ± SEM). HEK293 cells were used to test whether the receptor can also signal through cAMP. NKH-477 (a forskolin-analog), which activates adenylyl cyclase, will be responsible for an increase of intracellular cAMP levels. If the receptor couples negatively to adenylyl cyclase, a reduction of intracellular cAMP levels would be expected following administration of *Schgr*-AT. This was not observed when *Schgr*-ATR was expressed in the HEK293 cells (results not shown). However, in the absence of NKH-477, a specific increase of luciferase reporter activity was observed with an EC_50_ value of 8.10 × 10^−8^ M (Figure 4C; logEC_50_= −7.09 ± 0.123, mean ± SEM). CHO-WTA11, CHO-PAM28 and HEK293 cells transfected with an empty pcDNA 3.1 vector did not show any response to *Schgr*-AT (results not shown).

### Transcript level studies

**Figure 5:**
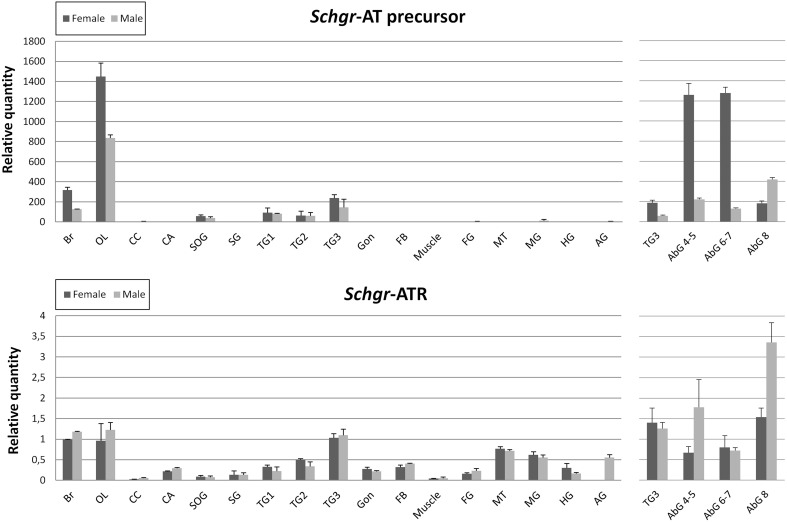
**Graphic representation of the transcript levels of the precursor of *Schgr*-AT and the *Schgr*-ATR measured in sexually mature *S. gregaria* in two experiments**. The data represent mean values ± S.E.M. of three independent tissue samples run in duplicate, normalized relative to Actin and GAPDH transcript levels. Abbreviations used: Br, brain; OL, optic lobes; CC, *corpora cardiaca*; CA, *corpora allata*; PG, prothoracic gland; SOG, suboesophageal ganglion; SG, salivary gland; TG1, prothoracic ganglion; TG2, mesothoracic ganglion; TG3, metathoracic ganglion; Gon, gonads; FB, fat body; Muscle, flight muscle; FG, foregut; MT, Malpighian tubules; MG, midgut; HG, hindgut; AG, male accessory glands, AbG, abdominal ganglia. The first three abdominal ganglia (1–3) are fused to the metathoracic ganglion (TG3). On the left the results of the first qRT-PCR study are depicted. The data of the gregarious and solitarious animals are represented together. On the right the results of the second qRT-PCR study are depicted.

The expression of the *Schgr-AT* precursor is largely restricted to the central nervous system (Figure 5). No significant differences were observed between samples of gregarious and solitarious animals, hence they are represented together. Females in general show higher *Schgr-AT* precursor transcript levels as compared to males. The effect is significant (*p* < 0.05) in the central brain parts, the optic lobes and abdominal ganglia 4-5 and 6-7. Only in the last abdominal ganglion the transcript levels are higher in the males than in the females (*p* < 0.05).

The *Schgr-ATR* is also mainly expressed in the CNS. The highest transcript levels can be measured in the brain, the optic lobes, the metathoracic ganglion and the abdominal ganglia (Figure 5). The receptor also shows relatively high transcript levels in the Malpighian tubules, intestine, male accessory glands, mesothoracic ganglion, prothoracic ganglion, fat body, gonads, CA and the salivary glands. No significant differences were observed between sexes, or phases. Nor did we observe significant differences between the transcript levels in larval and adult CA (results not shown). The *Schgr*-ATR transcript levels are 200–1000- fold lower in the central nervous system as compared to the *Schgr*-AT precursor transcript levels.

### Gut motility bio-assay

**Figure 6:**
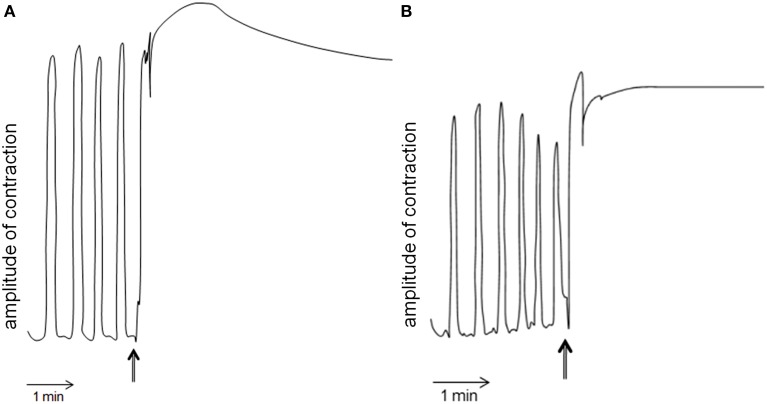
**Myotropic activity of *Schgr*-AT on the midgut of *Schistocerca gregaria***. Arrows indicate the administration of *Schgr*-AT. **(A)** and **(B)** represent two independent measurements on the same midgut.

*Schgr*-AT was added to the midgut preparation *in vitro* when a constant contraction rhythm was observed. This led to an immediate tetanus (Figure 6A left). After rinsing, the tetanus disappeared and normal contraction rhythm was restored (results not shown). No change in contraction strength or rhythm of the midgut was observed when we added saline without the peptide (results not shown). The entire procedure was repeated and again only change in contraction of the midgut could be observed when adding *Schgr*-AT (Figure 6B right).

### *In vitro* measurement of JH biosynthesis—radiochemical assay (RCA)

**Figure 7:**
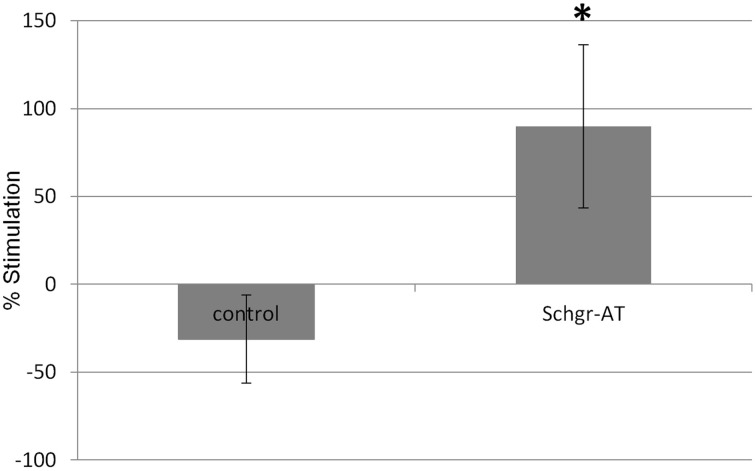
**The effect of *Schgr*-AT on the rate of JH biosynthesis in the CA as measured in an *in vitro* radio-chemical assay (RCA)**. The bars represent averages ± S.E.M. of 8 control and 9 *Schgr*-AT treated, individual CA dissected from vitellogenic adult females. Significant differences (*p* < 0.05) are indicated by an asterisk (^*^).

In the control CA the JH production was slightly lower during the second incubation period when compared to the first incubation. This is likely the result of the natural decrease in JH biosynthesis by senescence of the CA cells or a decrease in JH precursor pools as in the *in vitro* nature of the experiment. However, if the CA were treated with AT during the second incubation period, the JH production increased significantly (*p* < 0.05; Figure 7).

## Discussion

### Molecular cloning and phylogeny

In the present study, we have characterized *Schgr*-ATR, an AT receptor of the desert locust, *S. gregaria*. The obtained sequence has considerable similarity with orthologous receptors from other insects (Figure 1) (Horodyski et al., 2011; Vuerinckx et al., 2011; Nouzova et al., 2012; Verlinden et al., 2013). The sequence contains a DRW (instead of the typical DRY) motif in the second intracellular loop, as it is the case for other AT receptors. AT and its receptor seem to be present in both hemimetabolous and holometabolous insect species (Figure 3), although exceptions exist. *Drosophila* appears to lack both the ligand (Hewes and Taghert, 2001; Vanden Broeck, 2001) and its receptor. It was first thought that the hymenopteran insects, *A. mellifera* (Hummon et al., 2006) and *N. vitripennis* (Hauser et al., 2010) also lacked an AT-like peptide. However, after a more thorough search, a hymenopteran *AT* gene was found (Veenstra et al., 2012) and, meanwhile, an AT receptor was characterized in the hymenopteran species *B. terrestris* (Verlinden et al., 2013).

The ATRs show large sequence similarity to the mammalian orexin (ORX) receptors. Although the ORX and AT peptides do not display any obvious sequence similarity and ORX does not activate the invertebrate receptors (Vuerinckx et al., 2011), Mirabeau and Joly found evidence for a common origin of the AT and ORX precursor genes (Mirabeau and Joly, 2013).

### Functional receptor characterization

We demonstrated that *Schgr*-AT activates the *Schgr*-ATR *in vitro* and causes an increase in both intracellular calcium ion and cAMP levels with an EC_50_ value in the nanomolar range (Figure 4). The higher standard deviation measured in the HEK293 cells compared to the CHO cells can be explained by the absence of a normalization step for the total amount of cells per well, which was used in the calcium reporter assays. The measurement of the bioluminescence in the cAMP reporter assay is dependent on a CRE and thus dependent on the phosphorylation of CREB (cAMP Responsive Element Binding protein). Therefore, it has been speculated that a change in bioluminescence may also be caused by calcium, since CREB can also be phosphorylated by calcium/calmodulin-dependent protein kinase (Johannessen et al., 2004). However, an earlier study of another neuropeptide receptor (*Schgr*-sNPFR) that makes use of the same assays, showed increased intracellular calcium levels (CHO cell screen), but no increase in bioluminescence in the HEK293 cell screen, which would be expected if the calcium was responsible for the increase in bioluminescence in this screen (Dillen et al., 2013). In addition, the reporter plasmid was also used in various other studies (for example Hearn et al., 2002; Johnson et al., 2004). These facts suggest that an increase in bioluminescence in this assay is caused directly by an increase in intracellular cAMP levels and not (indirectly) by the increase in intracellular calcium levels.

Our pharmacological data correspond well with previous pharmacological characterizations of ATRs in other insects. Upon activation by their endogenous AT, the AT(L)Rs of *M. sexta*, *T. castaneum* and *B. terrestris* also stimulate intracellular calcium and cAMP levels, although lower EC_50_ values were measured (Horodyski et al., 2011; Vuerinckx et al., 2011; Verlinden et al., 2013). The fact that the EC_50_ value of the receptor expressed in HEK293 cells is higher than the EC_50_ value of the receptor expressed in CHO-PAM28 cells might indicate that the calcium response of *Schgr*-ATR is more sensitive than the cAMP response, although the difference may (in part) be explained by the use of different assays.

### Tissue distribution and functions of allatotropin

*Schgr*-AT precursor expression seems to be largely restricted to the central nervous system (Figure 5). This is as expected, since neuropeptides are usually produced in the nervous system and transported toward their target tissues (Caers et al., 2012). Our qRT-PCR data correspond very well with previous immunological and mass spectrometry data obtained in *L*. *migratoria* (Paemen et al., 1992) and *S. gregaria* (Homberg et al., 2004; Clynen and Schoofs, 2009) confirming the presence of this neuropeptide in extensive areas of the locust brain, including all neuropils in the optic lobe, the antennal lobes, and most areas in the protocerebrum. The first “AT-related” peptide in locusts was originally purified from male accessory glands of *L. migratoria* (*Lom*-AG-MT1) (Paemen et al., 1991) and is identical to *Schgr*-AT. The AT present in the male accessory glands (which show very low relative expression levels) is presumably originating from the (last) abdominal ganglia. Moreover, in other insect species specific neuroendocrine cells in the abdominal ganglia appear to be the most abundant source of AT (Veenstra et al., 1994, 2012; Veenstra and Costes, 1999; Rudwall et al., 2000; Neupert et al., 2009).

The *Schgr*-AT precursor shows 100–1000 fold higher transcript levels in the central nervous system as compared to the *Schgr*-ATR. This can be explained by the fact that neuropeptides are released in large quantities into the periphery, where they will bind to their receptors, to execute their functions. Moreover, the half-life of a peptide is expected to be shorter than the turnover rate of receptors.

The high abundance of ATR in the central nervous systems suggests a critical role in sensory processing, learning and memory and motor control (Elekonich and Horodyski, 2003). In *Leucophaea maderae*, injections of AT near the accessory medulla, which is identified as the location of the circadian clock in this cockroach and part of the optic lobes, resulted in changes in circadian locomotor activity (Petri et al., 2002). Also in *S. gregaria* high expression levels of ATR were measured in the optic lobes.

The ATR expression in the CA is probably related to the stimulatory role of AT on the biosynthesis and release of JH. Already, Tobe et al. (1977) demonstrated that an allatostimulatory factor released by the CA was responsible for the production of JH. We now also confirmed that *Sch*gr-AT indeed stimulates the JH production in the CA (Figure 7). This may also explain why the AT precursor expression is significantly higher in 10 day old adult females as compared to males (Figure 5), since JH (regulated by AT) was demonstrated to be important in females of this age for vitellogenin production and oocyte growth (Pratt and Tobe, 1974; Sevala et al., 1995; Glinka and Wyatt, 1996; Wyatt et al., 1996). The difference was especially pronounced in the abdominal ganglia, hence they could be responsible for activation of the CA.

The high expression levels of *Schgr*-ATR in the Malpighian tubules were also observed in *M. sexta* and suggest that AT may have a role in osmoregulation (Horodyski et al., 2011). The expression of the *Schgr*-ATR in the salivary glands can be related to a role in the stimulation of saliva secretion as was recently discovered in *R. prolixus* (Masood and Orchard, 2014). High transcript levels of *Schgr*-ATR were measured in the digestive system as well. This can be explained by the fact that AT affects the intestinal motility, as was shown with the bio-assay (Figure 6). Another link that can be made with the digestive system, is the impact of the nutritional status on the transcript levels of the AT precursor. In larvae of *M*. *sexta* and the armyworm *Mythimna separate* it was shown that starvation led to higher transcript levels of AT (Lee and Horodyski, 2002, 2006; Zhang et al., 2008). In larvae, starvation causes an additional molt, indicating that JH levels are elevated. This gives the larvae the opportunity to acquire additional nutrients once they become available in order to successfully complete development to a robust reproductive adult. In contrast, starvation of some insects during the adult stage inhibited oocyte maturation as a consequence of decreasing JH biosynthesis (Tobe and Chapman, 1979; Zhang et al., 2008). The overall regulation of JH titer is complex, since the CA can be influenced by multiple stimulatory and inhibitory factors, and since JH catabolism and binding to JH transport proteins also plays a major role in the control of JH titer (Lee and Horodyski, 2006).

## Author contributions

Molecular cloning and EST database analyses were performed by LB, PV, HV and EL. Functional tests with AT were performed by PV, HV, CL, RV and SZ. The radio chemical assay was performed by EM and ST. The dissections were performed by EM, LB and HV. The pharmacological characterization was performed by EL. Guidance of the study, writing and correction of the manuscript were performed by HV, EL and JV.

### Conflict of interest statement

The authors declare that the research was conducted in the absence of any commercial or financial relationships that could be construed as a potential conflict of interest.
