# Supplementary material for: Molecular cloning and characterization of the allatotropin precursor and receptor in the desert locust, Schistocerca gregaria
Source: Front Neurosci. 2015 Mar 12;9:84. doi: 10.3389/fnins.2015.00084 (PMC4357254; doi:10.3389/fnins.2015.00084)
Supplement: Supplementary file 1 [file Table1.DOCX]

Supplementary Table 1. Gene specific oligonucleotide primers used for RACE of *Schgr*-ATR in this study.

|  | 5' RACE | 3' RACE |
| --- | --- | --- |
| cDNA synthesis | 5'-GTACACCATGTTCCAGTAGT-3' | 5'-CATGTTCGCCATCTGCTAC-3' |
| PCR 1 | 5'-CCAGAGAGAAGTCTGGAGTG-3' | 5'-TCCACCTGCTCAACATACTG-3' |
| PCR 2 | 5'-ACATTGGAGGACGGTGACA-3' | 5'-CCGTCAACCCAGTCATCTAC-3' |
