# Supplementary figures and images for: Molecular cloning and characterization of the allatotropin precursor and receptor in the desert locust, Schistocerca gregaria
Source: Front Neurosci. 2015 Mar 12;9:84. doi: 10.3389/fnins.2015.00084 (PMC4357254; doi:10.3389/fnins.2015.00084)

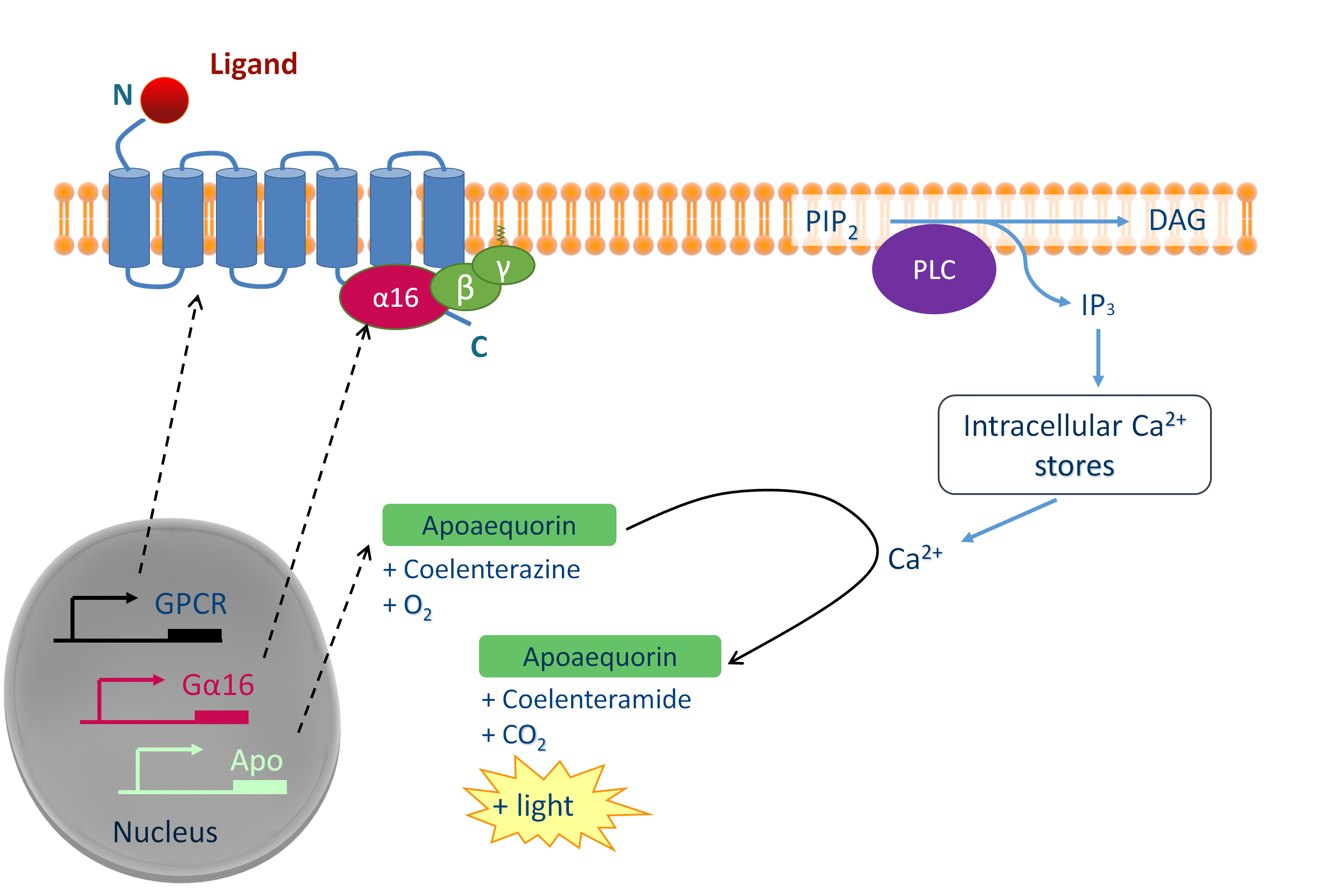

Supplement: Supplementary file 2 [file Image1.TIF]

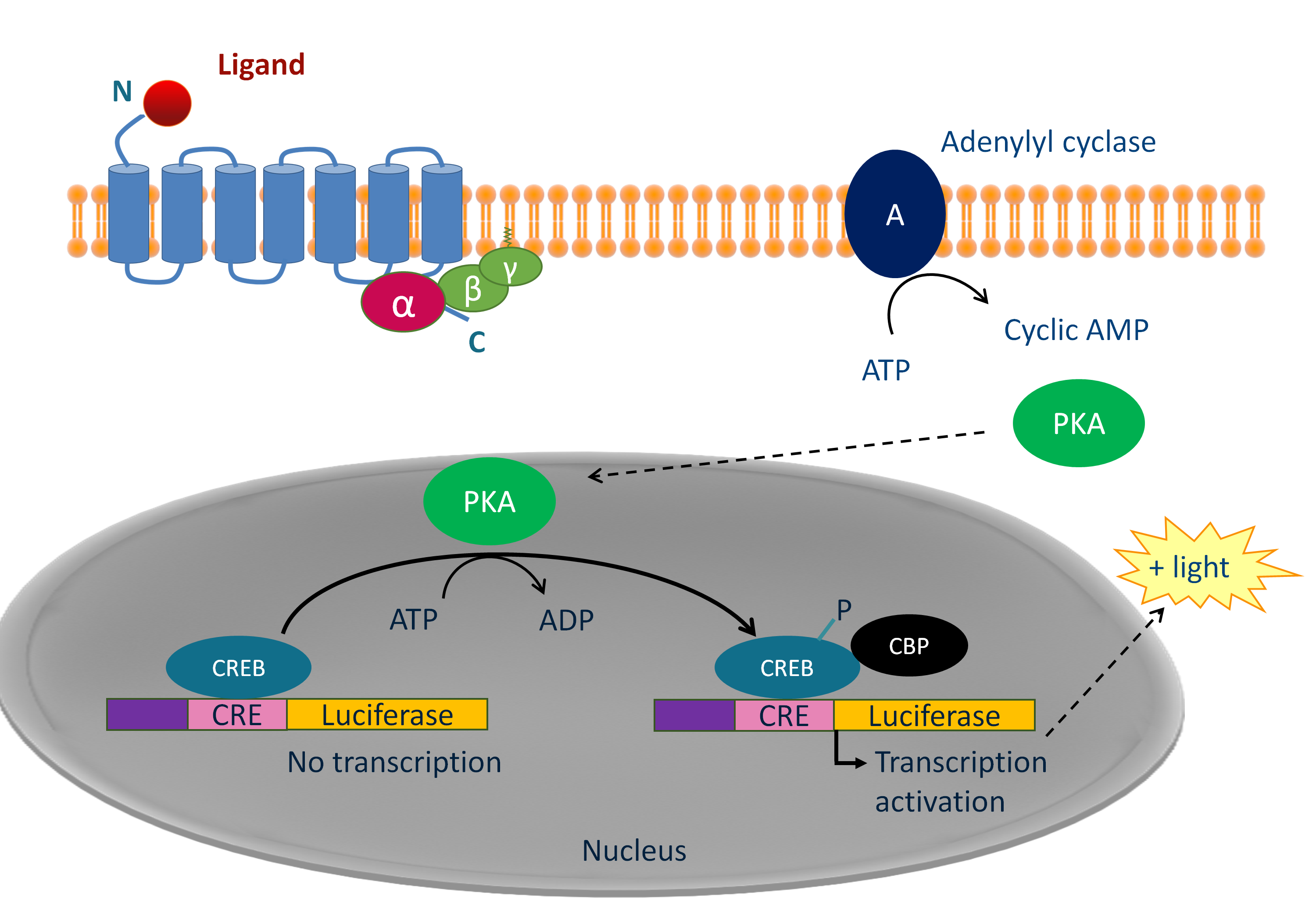

Supplement: Supplementary file 3 [file Image2.TIF]

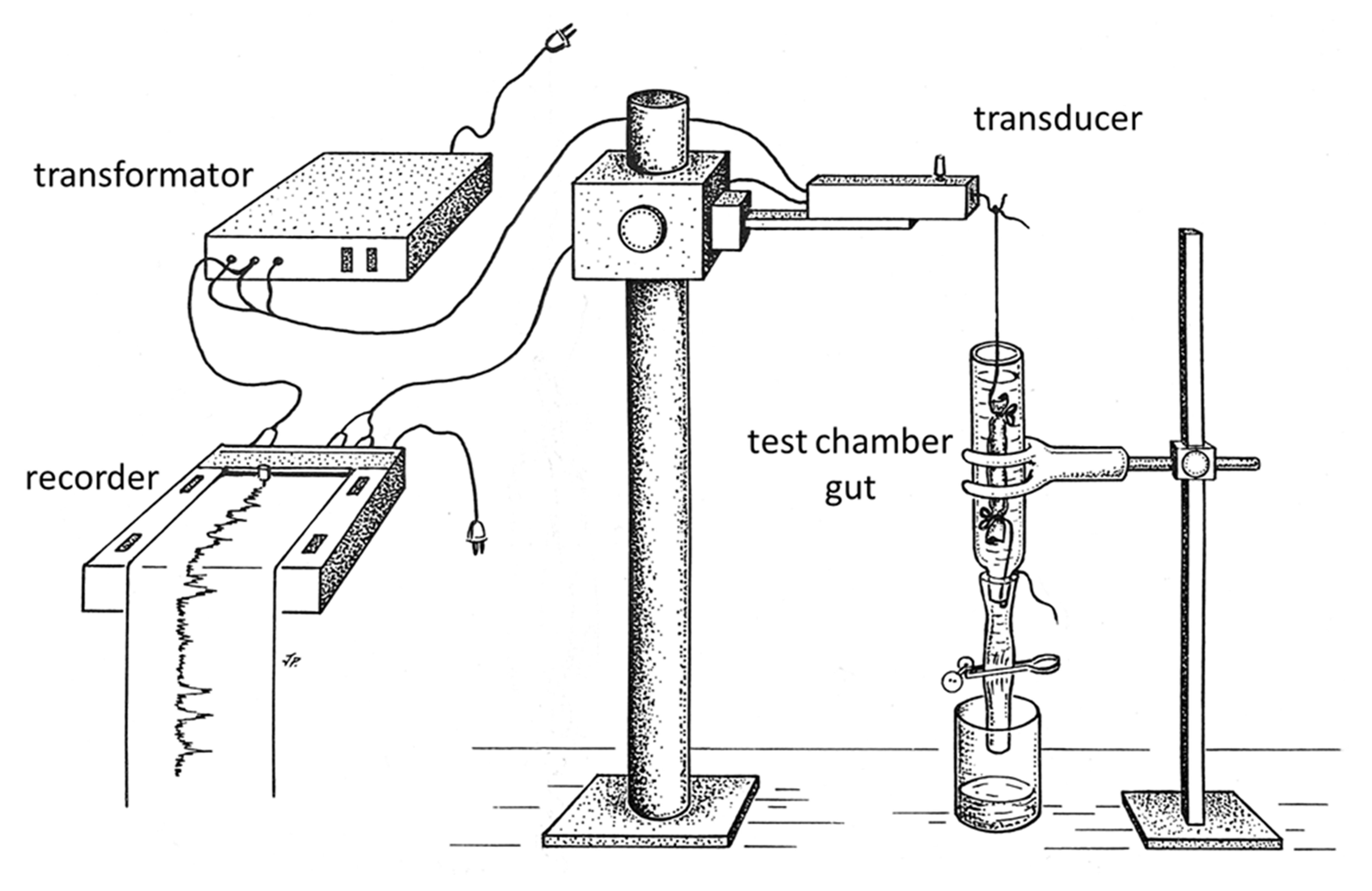

Supplement: Supplementary file 4 [file Image3.TIF]
